# Evidence of Season-Dependency in Vegetation Effects on Macrofauna in Temperate Seagrass Meadows (Baltic Sea)

**DOI:** 10.1371/journal.pone.0100788

**Published:** 2014-07-07

**Authors:** Maria Włodarska-Kowalczuk, Emilia Jankowska, Lech Kotwicki, Piotr Balazy

**Affiliations:** Department of Marine Ecology, Institute of Oceanology Polish Academy of Sciences, Sopot, Poland; Seagrass Ecosystem Research Group, Swansea University, United Kingdom

## Abstract

Seagrasses and associated macrophytes are important components of coastal systems as ecosystem engineers, habitat formers, and providers of food and shelter for other organisms. The positive impacts of seagrass vegetation on zoobenthic abundance and diversity (as compared to bare sands) are well documented, but only in surveys performed in summer, which is the season of maximum canopy development. Here we present the results of the first study of the relationship between the seasonal variability of seagrass vegetation and persistence and magnitude of contrasts in faunal communities between vegetated and bare sediments. The composition, abundance, biomass, and diversity of macrozoobenthos in both habitats were compared five times throughout the year in temperate eelgrass meadows in the southern Baltic Sea. Significant positive effects of macrophyte cover on invertebrate density and biomass were recorded only in June, July, and October when the seagrass canopy was relatively well developed. The effects of vegetation cover on faunal species richness, diversity, and composition persisted throughout the year, but the magnitude of these effects varied seasonally and followed changes in macrophyte biomass. The strongest effects were observed in July and coincided with maximums in seagrass biomass and the diversity and biomass of other macrophytes. These observations indicate that in temperate, clearly seasonal systems the assessment of macrophyte impact cannot be based solely on observations performed in just one season, especially when that season is the one in which macrophyte growth is at its maximum. The widely held belief that macrophyte cover strongly influences benthic fauna in marine coastal habitats, which is based on summer surveys, should be revisited and complemented with information obtained in other seasons.

## Introduction

Seagrasses and associated macrophytes are important structural and functional components of coastal ecosystems in temperate and tropical seas. They are classic examples of marine ecosystem engineers [1], as they can modify water flow regimes and sedimentation rates, and, hence, modulate the availability of resources to other species [2]. They play the important role of habitat-forming species both under ground, with root systems, and above ground, with vegetative organs, which contribute three-dimensional structures to the sea bed architecture and provide shelter and numerous niches for other organisms, mostly benthic invertebrate and fish species [3]. Seagrasses and associated macrophytes, especially epiphytes, can be direct food sources and sustain a number of grazing invertebrates; thus, they shape carbon flow pathways in shallow-water food webs [4]. The effects of seagrass vegetation on the other components of the benthic systems can be best perceived when seagrass meadows and other habitats are compared [5]. Several studies compare the zoobenthic communities in vegetated and bare sediments and document the positive impact of seagrass vegetation in terms of density, biomass, and diversity [6]-[8]. However, the vast majority of reports on the positive impacts of seagrasses on benthic fauna are based on results of surveys conducted only in summer when seagrass meadows are logistically most accessible but also when seagrass canopies are at their maximum of annual development. To our knowledge, there have been almost no attempts to explore the persistence of these effects in other seasons. The only report of seasonal contrasts in zoobenthic response to vegetation is Gambi et al. [9], who compare polychaete assemblages in *Posidonia oceanica* and *Zostera noltii* vegetated sediments and on bare sand in the Mediterranean Sea based on a study performed in two seasons - summer and winter. Some indication of seasonality in the importance of seagrass vegetation for associated fauna is reported by Berkenbusch et al. [10]. They identify the biomass of *Zostera novazelandica* as the best explaining factor for macrofaunal community structure in summer at the seagrass growing optimum, while it is much less important in fall. The lack of the seasonal aspect in the rich literature on seagrass influences on zoobenthic communities is a significant gap in understanding the functioning of these systems.

Ecosystem engineer effects can depend on the quantitative characteristics of engineering species populations [1], [11], [12]. Thus, it can be expected that strong seasonality in these populations can influence their role in local systems. Fortino [13] explores the effects of benthivorous fish on sediment accumulation in a North American river, but, upon finding no effect in winter, linked it to temperature-driven seasonal reductions in fish activity levels. Berkenbusch et al. [10] report that the bioturbation effects of the ghost shrimp *Callianassa filholi* on intertidal flats persisted throughout the year, but the magnitude of impact varied seasonally correspondingly to changes in bioturbator density. Regarding seagrasses, Hasegawa et al. [14] show that the occurrence and magnitude of the engineering effects of *Zostera marina* (e.g., reduction of current velocity, buffering of sediment resuspension) was season dependent and followed temporal changes in seagrass canopy development. These reports support Fortino's [13] claim that the quantification of engineering organism effects during a single season may exaggerate, or downplay, the role of these processes throughout the year, and a comprehensive assessment of the significance of a given species must be based on information obtained in all seasons.

Seagrass and associated macrophyte canopies can vary substantially in annual cycles [15]. Seasonal variability is generally greater at higher latitudes [16], but clear seasonal trends can be also observed at lower latitudes (e.g. [17]). Duarte [16] analyzed annual biomass variability of 11 macrophyte species, and reports that the coefficient of variation (i.e., variability above the annual mean) ranged from 17% to 120% and was dependent on latitude, but it could also vary locally and with depth and exposure to disturbance. Seasonal dynamics are also species-specific with stronger interannual variability in smaller species (e.g., it is more pronounced in *Z. marina* compared to *P. oceanica*; [18]). Several populations of *Z. marina* in northwest Europe and North America were even reported to employ annual or semi-annual life histories; when no or only a small percentage of vegetative shoots survive winter, and the persistence of a population relies on extensive seed production and new recruitment each spring [19], [20]. In some regions, knowledge of the seasonal variability of macrophyte vegetation in seagrass meadows is lacking or very poor. For example, in the Baltic Sea there have been no reports on the status of seagrass vegetation in different seasons, except for a recent study by Jankowska et al. [21] that was performed in the Puck Bay, which is located in Polish coastal waters.

Duarte [16] suggests that assessing the importance of seagrass at its maximum biomass may not paint a complete picture of its role in systems. Therefore, quantifying biomass variability is important if we are to evaluate the role of seagrasses in coastal ecosystems. Temporal variability in seagrass biomass is likely to determine temporal variability in habitat structure or food availability for the associated biota. Duarte [16] also predicted that latitude-related change in degrees of seasonality can have important ecosystem implications. At low latitudes, benthic communities associated with seagrass beds can benefit from stable levels of macrophyte nutritional and structural support, while in temperate waters, fauna experience considerable seasonal habitat change that can influence its functional and structural balance throughout the year. However, this hypothesis has never been tested.

Jankowska et al. [21] offers the first report on the seasonality of macrophyte cover in Baltic Sea eelgrass meadows. These authors demonstrate that seagrass vegetation persists throughout the year, but both shoot density and the aboveground biomass of the plant as well as the abundance and diversity of associated macrophytes undergo substantial seasonal changes with clear declines in winter. It is intriguing to learn if the seasonality in macrophyte vegetation shapes the patterns of differences in other components of benthic ecosystems between vegetated and bare sediments. Based on the results of other studies performed in Baltic Sea eelgrass meadows [6], [22], we can anticipate that a strong positive impact of macrophyte vegetation on zoobenthic composition, abundance, and diversity occurs in summer, but we do not know if these effects persist in seasons when the macrophyte canopy is not at its maximum. In the present study we aimed to test: 1) if differences in composition, abundance, biomass, and diversity of macrozoobenthic communities between vegetated sediments and bare sand in southern Baltic *Z. marina* meadows are consistent across different seasons; 2) if the persistence and magnitude of these differences mirror seasonal changes in macrophyte cover quantitative and qualitative characteristics. In this paper, we present the first analyses of the relationship between the temporal variability of macrophyte vegetation in a seagrass meadow and its impacts on associated fauna.

## Material and Methods

### Ethics Statement

The collection of macrophytes, sediment and macrofauna samples undertaken for this research was approved by collection permits issued to a “ZOSTERA. Restitution of key elements of the inner Puck Bay ecosystem,” project (no. POIS.05.01.00-00-205/09-00) coordinated by Center of Coordination of Environmental Projects and Regional Directorate of Environmental Protection and Nature Conservation at the Pomeranian Voivodship.

### Sampling and laboratory analyses

The samples were collected in the Puck Bay, which is a shallow embayment in the southwestern part of the Gulf of Gdańsk (Baltic Sea) separated from the open sea by the Hel Peninsula. The eight-kilometer Ryf Mew sandbank divides the bay into two parts: the inner Puck Lagoon and the outer Puck Bay. The depths and bottom morphology of these two parts of the bay differ. The outer Puck Bay is notably deeper at an average depth of 20.5 m, while the Puck Lagoon is considerably shallower with an average depth of 3.1 m [23]. Extensive *Zostera marina* meadows were present in the Puck Lagoon and the outer Puck Bay in the 1950s, but from the late 1950s to the 1980s degradation of the meadows and the gradual replacement of eelgrass by filamentous algae and *Zanichella palustris* was observed [24]. A recent inventory of the Polish Exclusive Economic Zone sea bed habitats documented that areas covered by *Z. marina* meadows are growing in size [25].

**Figure 1:**
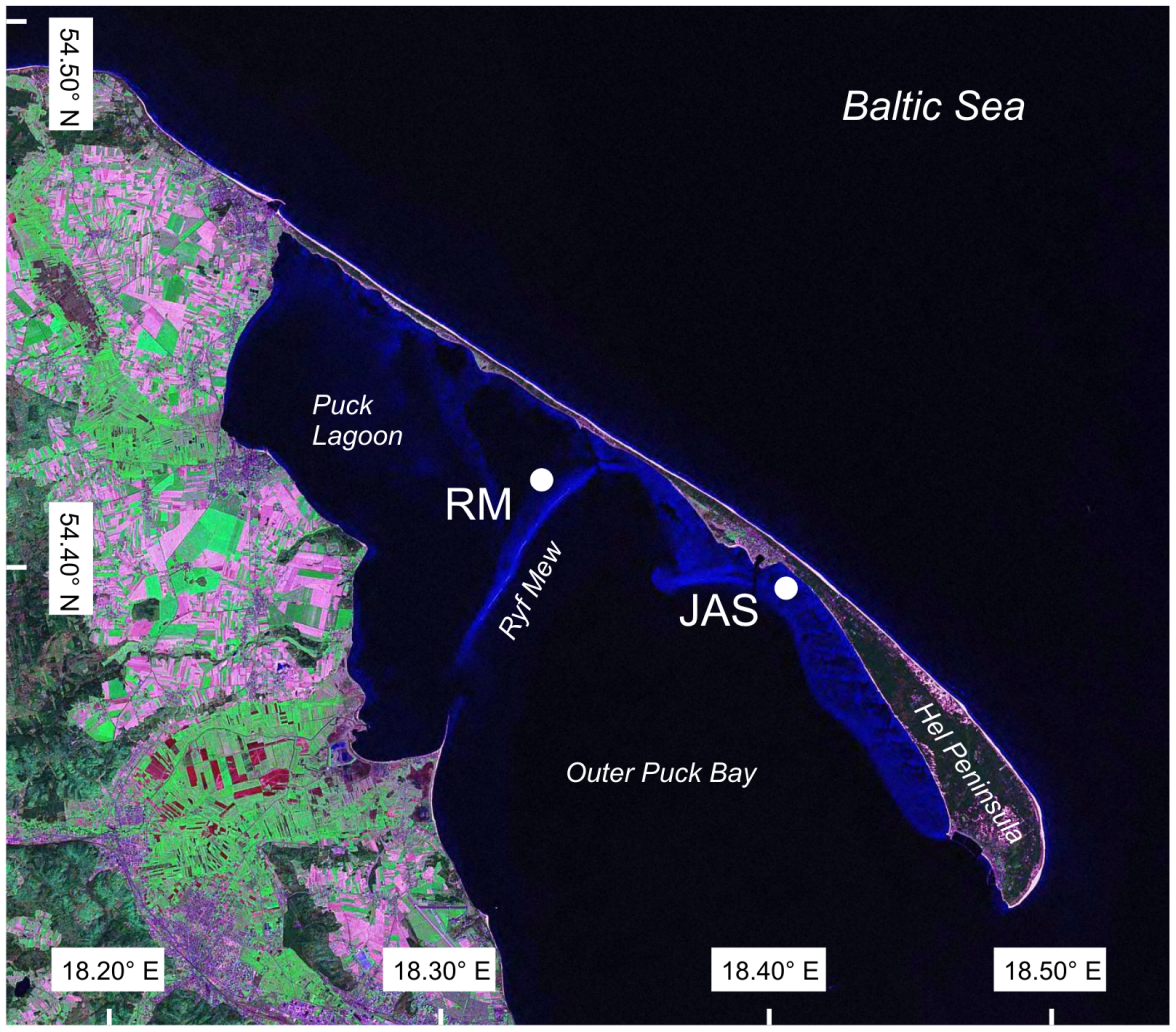
Location of sampling sites in the Puck Bay. The map is based on Landsat picture taken in 2000, publicized by NASA and located in public domain of Wikipedia (http://en.wikipedia.org/wiki/File:Mierzeja_Helska.jpg).

Two localities were selected for sampling: 1) RM – located in Puck Lagoon close to the shallows of the Ryf Mew sandbank at a depth of 3 m (54°42,7 N 18°33,7 E); 2) JAS – located in the outer part of the Puck Bay east of Jastarnia at a depth of 1.5 m (N 54°41,4 E 18°40,9; Figure 1). Samples were collected five times throughout the year from October 2010 to October 2011. Sampling started in fall 2010 (10–13 October 2010, OCT'). Sampling representative of winter conditions was performed on 30–31 March 2011 (MAR) just after the ice cover disappeared. The winter of 2010/2011 was very cold, and the Puck Bay was covered with ice until 20 March (http://www.smhi.se/oceanografi/iceservice/is_prod_en.php). However, high concentrations of chlorophyll a in the surface sediments at this sampling time [21] suggested that the diatom bloom could have already occurred; thus, based on the pelagic system phenology, this sampling should rather be defined as having been performed in early spring. Regarding the macrophytes, the values of species richness, density and biomass were very low – indicating that vegetation still remained in the winter phase of development. The samples were then collected in late spring (1 June 2011, JUN), summer (19 July 2011, JUL), and fall (8 and 15 October 2011, OCT).

At each sampling location five replicate sets of samples were collected from the vegetated seabed and bare sand. Each set of samples included one collected with a 15 cm diameter large core for macrozoobenthos and three sediment samples collected with a 2 cm diameter small core. Sediment samples were collected to analyze grain size (upper 10 cm), POC and TN concentrations, δ^13^C, δ^15^N (upper 2 cm), and photosynthetic pigment concentrations (upper 2 cm). The samples for macrofauna were sieved through 0.5 mm mesh and then fixed in 4% formaldehyde. The sediment samples for photosynthetic pigment analyses were frozen at −80°C.

In the laboratory, chlorophyll a and pheopigment concentrations were assessed with the spectrophotometric method [26]. POC, TN, δ^15^N, and δ^ 13^C in sediments were analyzed using an Elemental Analyzer Flash EA 1112 Series combined with an Isotopic Ratio Mass Spectrometer IRMS Delta V Advantage (Thermo Electron Corp., Germany). To determine the grain-size distribution, sediment samples were dried (48 h, 60°C) and sieved through mesh sizes at thirteen 0.5 phi intervals [27]. In the laboratory all macrofaunal individuals were identified to the level of species or the lowest possible taxonomic level, counted, and weighed. Macrophytes were identified to the lowest possible taxonomic level. Algae and plants were dried in 60°C for 48h and weighed. Seagrass shoots were counted, leaf length was measured, and the dry weight of the aboveground and the belowground parts was determined.

### Data analyses

Species richness, defined as the number of taxa in a sample (*S*), species diversity measured with the Shannon-Wiener diversity loge based index (*H*), and evenness of distribution of individuals among taxa expressed by the Pielou index (*J*), were calculated for all macrobenthic samples. Differences in macrozoobenthic univariate characteristics (density, biomass, *S*, *J*, *H*) among the five months (Mt) and between the two stations (St), and two bottom types (Bt, vegetated bottom and bare sand) were tested using the three-way PERMANOVA model based on a similarity matrix created from the Euclidean distances among samples. The PERMANOVA routine does not rely on the assumption of normally distributed data and uses permutations to test null hypotheses. Unbiased estimates of each of the components of variation (CV) were calculated from mean squares to compare the amount of variation that is attributable to different terms in the model. When a significant effect of Bt and significant interaction of BtxMt were detected, pairwise tests for differences between bottom types within the five months were performed. The magnitude of the effect of vegetation on macrofauna univariate characteristics in different months was estimated using of the veg/bare ratio, which is the ratio of the mean recorded on vegetated bottom to that on bare sand.

Bray-Curtis similarities were calculated for presence/absence data (pr/ab), double root transformed, and untransformed data (nonTR) of macrobenthic species abundances in the samples. The three-way PERMANOVA model, with the three factors of Mt, St, and Bt, was applied to the similarity matrices. The patterns of macrobenthic composition were visualized with PCO plot. In addition, CAP (constrained ordination, discriminating among *a priori* groups) was used to visualize the variability along the two axes that best discriminated groups of samples defined by bottom type and month. When the significant effect of Bt and the significant interaction of BtxMt was detected by the main PERMANOVA test, pairwise tests for differences between bottom types in the five months were performed. The magnitude of the effects of vegetation on the macrobenthic community composition in different months was estimated with the distance (Bray Curtis index) between centroids representing the fauna collected from two bottom types. An nMDS plot of Bray-Curtis similarities between averaged species abundances in groups of samples defined by two sites, two bottom types, and five months was constructed to visualize multivariate variability among these groups of samples.

Relationships between environmental variables and macrobenthic community composition were investigated using the Distance-based Linear Models (DISTLM) procedure in PERMANOVA+ [28]. The original data set of environmental variables included 13 quantitative and three categorical (nominal) variables (Mt, St, Bt, as defined above). Quantitative variables included descriptors of organic matter content (δ^13^C, δ^15^N, POC, TN, POC/TN), photosynthetic pigments (chla - concentration of chlorophyll a, CPE – chloroplastic pigment equivalents, i.e., the concentration of all photosynthetic pigments, including pheopigments, %chla – percentage of chla in CPE, chla/POC), and the granulometric characteristics of sediments (mean grain size, sorting, fine sand and coarse sand fractions). These variables were tested preliminarily for collinearity using Draftsman plot and the Spearman correlation matrix. Based on the results, only six quantitative variables were left for further analysis (POC, POC/TN, chla, δ^13^C, mean grain size, fine sand). The forward selection procedure was used to determine the best combination of predictor variables [28].

For samples collected on vegetated bottom the DISTLM analyses was used to explore the relationships between macrophyte biometrics and macrozoobenthic community characteristics. The original data set included 6 quantitative variables: total macrophyte biomass, seagrass above ground biomass, total seagrass biomass, seagrass shoot density, algal biomass and algal species richness. These variables were tested preliminarily for collinearity using Draftsman plot and the Spearman correlation matrix. Based on the results, seagrass total biomass was excluded from further analysis. Similarity matrix created from the Euclidean distances among samples was used for macrozoobenthic univariate characteristics (density, biomass, species richness) while Bray-Curtis similarities of double-root transformed data of species abundances in samples were used for the analyses focused on faunal species composition. The forward selection procedure was used to determine the best combination of predictor variables.

The SIMPER procedure was applied to identify species that were responsible for differences in macrobenthic communities between bare sand and vegetated bottom. SIMPER analysis was performed on Bray-Curtis similarities of both double-root transformed and presence absence data. The SIMPER procedure is based on the breakdown of average dissimilarity between groups into separate contributions from each species [29]. The average contribution to the overall dissimilarity divided by standard deviation (diss/SD) and the percentage of this contribution to total dissimilarity (contr%) were calculated and used to identify discriminating species.

## Results

**Figure 2:**
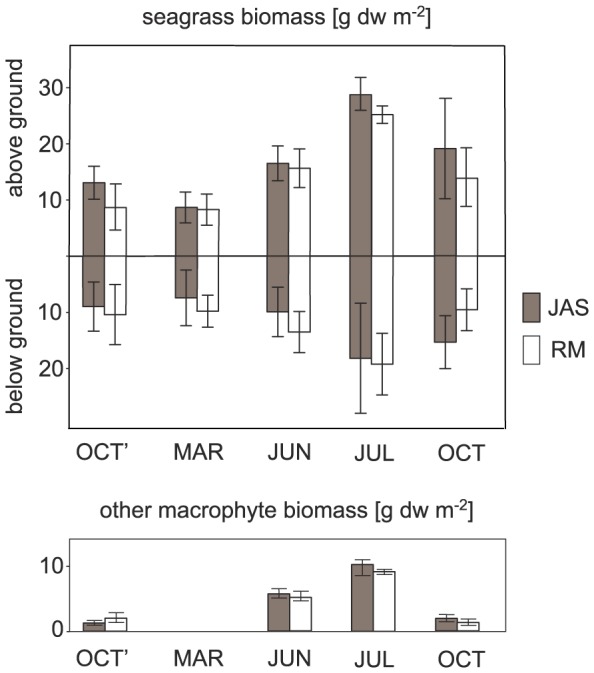
Seasonal variability in macrophyte vegetation characteristics. Seagrass aboveground and underground biomass and other macrophyte biomass [g dw m^−2^] recorded in 5 months (OCT' - October 2010, MAR - March 2011, JUN - June 2011, JUL - July 2011, OCT - October 2011) at two sampling stations (JAS - gray bars, RM - white bars). Mean and 0.95 CI values are presented. Based on data published by Jankowska et al. [20].

Similar seasonal patterns were observed for all measured seagrass vegetation characteristics (seagrass density, aboveground and belowground biomass, leaf length) with minimum values recorded in MAR and maximum in JUL (Figure 2). The biomass and diversity of other macrophytes also differed among the months studied. In MAR, no macrophyte species other than *Z. marina* was recorded. The highest biomass and species diversity (six species) was recorded in JUL. Brown filamentous algae *Pilayella litoralis* comprised from 30% to 80% of the total biomass of macrophytes accompanying eelgrass in this meadow. Further details of macrophyte vegetation trait variability at the studied stations are reported by Jankowska et al. [21].

**Figure 3:**
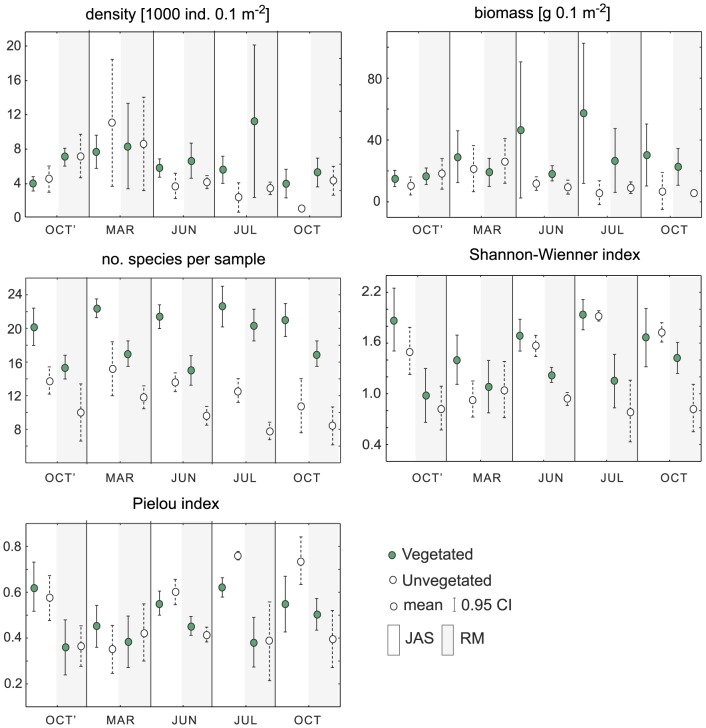
Seasonal variability in macrozoobenthic community characteristics. Density [1000 ind. 0.1 m^−2^], biomass [g 0.1 m^−2^], species richness (number of species per sample), diversity (Shannon-Wiener index), and evenness (Pielou index) recorded in 5 months (OCT'- October 2010, MAR - March 2011, JUN - June 2011, JUL - July 2011, OCT - October 2011) at two stations (JAS - white background, RM - gray background) and two bottom types (vegetated, unvegetated). Mean and 0.95 CI values are presented.

**Figure 4:**
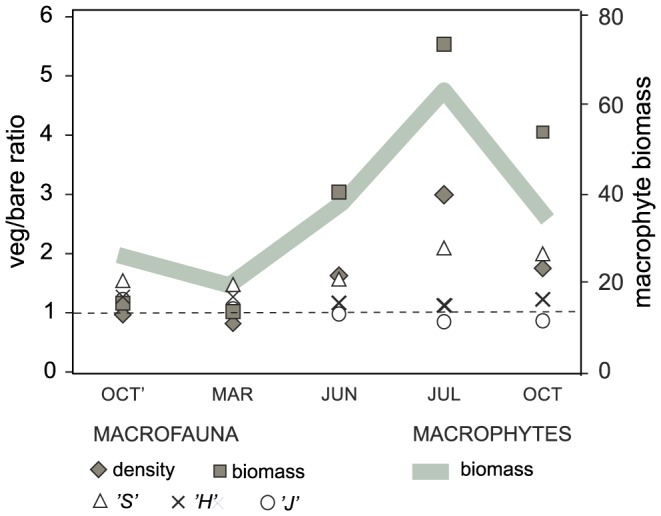
Magnitude of difference in macrofauna univariate characteristics between two bottom types plotted against total macrophyte biomass [g dw m^−2^]. Veg/bare ratio - ratio of mean recorded on vegetated bottom to mean on bare sand, for samples collected in 5 months (OCT'- October 2010, MAR - March 2011, JUN - June 2011, JUL - July 2011, OCT - October 2011). Veg/bare ratio presented for density, biomass, number of species per sample (*‘S’*), Shannon-Wiener index (*‘H’*), Pielou index (*‘J’*).

There were significant differences in macrobenthic density among months, stations, and bottom types (PERMANOVA, P<0.05, Table 1). The lowest mean density was recorded in OCT on bare sand at the JAS site (1002.2±150.1 SD ind. 0.1 m^−2^), the highest mean density and high variability was observed in JUL on the vegetated bottom at the RM site (11003.5±7144.6 SD ind. 0.1 m^−2^, Figure 3). There was significant interaction between month and bottom type, and pairwise tests identified significant effects of vegetation on macrofauna density only in the three months of JUN, JUL, and OCT (Table 2). The strongest effect was documented in JUL, when the mean density on the vegetated bottom was almost three times higher than that recorded on bare sand (Figure 4). Macrobenthic biomass differed significantly between the two stations and two bottom types (PERMANOVA, P<0.05, Table 1), while it did not differ among the sampling months. The lowest mean biomass was recorded in OCT on bare sand at the RM site (5.8±1.1 g 0.1 m^−2^). The very high mean biomass and highest variability in values were recorded in JUN and JUL in vegetated sediment at the JAS site (46.5±35.4 and 57.2±36.5 g 0.1 m^−2^, respectively). The factor of bottom type had the strongest effect on macrobenthic biomass (the highest CV value, Table 1), but this influence was dependent on month (significant MtxBt interaction, Table 1). The positive effect of vegetation was observed only in JUN, JUL, and OCT, with more than a fivefold increase in faunal biomass on the vegetated bottom documented in JUL (Figure 4).

**Table 1:** Three-way PERMANOVA tests for differences in macrofauna univariate characteristics among stations (St), months (Mt) and bottom types (Bt).

		density		biomass		*S*		*H*		*J*	
source	df	PsF	CV	PsF	CV	PsF	CV	PsF	CV	PsF	CV
St	4	9.4*	359.9	4.8*	3.9	166.7**	2.9	208.1**	0.4	125.6**	0.1
Mt	1	10.2**	595.0	1.9	3.1	6.6**	0.8	7.9**	0.1	10.9**	0.0
**Bt**	**1**	7.9*	**326.0**	30.2**	**10.5**	607.0**	**5.6**	32.1**	**0.2**	**0.7**	**0.0**
StxMt	4	2.1*	293.8	1.7	3.7	1.2	0.2	12.2**	0.2	11.1**	0.1
StxBt	4	1.5	122.7	9.5*	8.3	1.9	0.3	1.5	0.0	4.2*	0.0
**MtxBt**	**1**	5.4**	**579.8**	5.2**	**9.3**	11.0**	**1.6**	**0.2**	**0.0**	**1.5**	**0.0**
MtxStxBt	4	1.2	141.3	0.9	−2.0	2.0	0.7	5.6**	0.2	5.9**	0.1
Res	80		876.1		14.2		1.6		0.2		0.1
Bt (MtxBt)		Jun, Jul, Oct		Jun, Jul, Oct		all		all		—	

Results of tests for density, biomass, number of taxa per sample (*S*), Shannon-Wienner index (*H*), and Pielou index (*J*) are presented. Effects of bottom type and MtxBt interaction printed in bold. PsF - PERMANOVA pseudoF, CV - component of variation. Significant effects: * P<0.05, ** P<0.001. Bt (MtxBt) - significant effects in pairwise tests for differences between vegetated and unvegetated bottoms performed separately for five months.

**Table 2:** Three-way PERMANOVA tests for differences in macrobenthic community among stations (St), month (Mt) and bottom type (Bt).

		pr/ab		dbrt		nonTr	
source	df	PsF	CV	PsF	CV	PsF	CV
St	4	39.1**	12.1	45.4**	13.0	34.1**	17.8
Mt	1	12.0**	10.4	13.7**	11.0	10.1**	14.8
**Bt**	**1**	39.1**	**20.9**	92.9**	**18.7**	17.1**	**12.4**
StxMt	4	4.0**	7.7	4.9**	8.6	6.6**	16.3
StxBt	4	4.0*	4.8	5.1**	5.6	7.8**	11.4
**MtxBt**	**1**	2.9*	**6.1**	5.8**	**9.5**	5.7**	**15.1**
Mt x St x Bt	4	1.6	4.9	2.8**	8.2	3.9**	16.6
Res	80		13.9		13.8		21.8
Bt (MtxBt)		all		all		Oct', Jun, Jul, Oct	

Tests performed on Bray-Curtis similarity matrices for: presence/absence data (pr/ab), double root transformed (dbrt), and untransformed data (nonTr). Effects of bottom type and MtxBt interaction printed in bold. PsF - PERMANOVA pseudoF, CV - component of variation. Significant effects: * P<0.05, ** P<0.001. Bt (MtxBt) - significant effects in pairwise tests for differences between vegetated and unvegetated bottoms performed separately for five months.

Forty six taxa were identified in samples (Table S1). Species richness per sample (*S*) differed significantly among months, bottom types, and stations (PERMANOVA, P<0.05, Table 1). The three-way PERMANOVA pseudoF values indicated that bottom type and station had a much stronger effect on species richness than did month (Table 1). Throughout the year *S* was higher at the JAS site (mean 17.4±4.6 SD) than at the RM site (mean 13.2±4.3, Figure 3). *S* was also higher in vegetated bottoms than on bare sand for all combinations of months and sampling sites. The effect of vegetation on *S* was significant in all months, but the ratio of the mean recorded in vegetated sediments to that on bare sand increased from 1.5 in OCT' and MAR and 1.6 in JUN to 1.9 in OCT and 2.1 in JUL (Figure 4). Evenness (*J*) was higher in samples collected at the JAS site than at RM, but this difference was not consistent throughout the sampling period as was indicated by the significant effects of months and significant MtxSt interaction (Table 1). The lowest and highest mean values of *J* were recorded on bare sand at the JAS site, with the lowest in March (0.351±0.084 SD) and the highest in July (0.760±0.015, Figure 3). PERMANOVA did not detect any significant effect of bottom type on *J* (Table 1). Station, month, and bottom type were sources of significant difference in species diversity (*H*), and station had the strongest effect (as indicated by the highest CV). *H* at the JAS site was 1.62 on average (±0.33 SD), while at RM it was 1.03 (±0.27 SD). *H* was significantly higher on vegetated bottoms than on bare sand, but this effect did not interact with the effect of month (Table 1).

**Figure 5:**
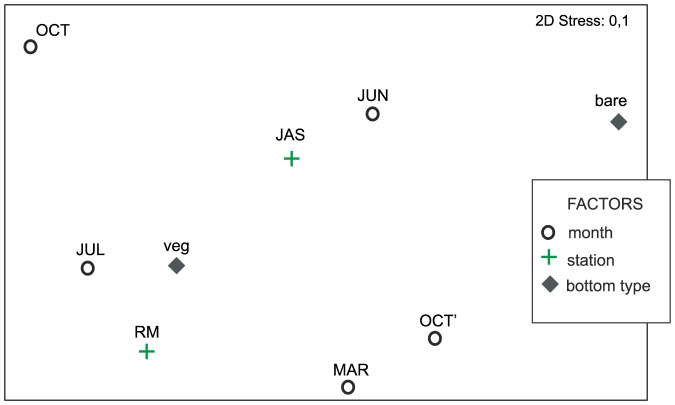
nMDS plots of Bray-Curtis similarities of average species abundances computed for groups of samples representing months, stations, bottom types. Data were double root transformed.

**Figure 6:**
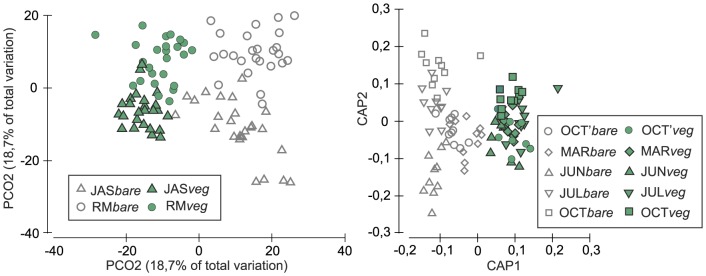
PCO of Bray-Curtis similarities of macrobenthic species abundances in samples (left) and CAP - ordination best discriminating groups of samples defined by bottom types and months (right). Data were double root transformed. Symbols represent sites/bottom types (left) and months/bottom types (right).

PERMANOVA identified significant effects (at P<0.001) of Bt, St, and Mt on macrobenthic community composition (Table 2). Bottom type had the strongest effect when pr/ab or double root transformed data were analyzed, as indicated by the highest CV values and illustrated on the MDS plot of group averages (Figure 5). Groups defined by bottom type and site could be easily discriminated on the PCO plot (Figure 6). Samples collected on vegetated bottoms and on bare sand were also clearly separated on the (St, Mt) CAP plot (Figure 6). On this plot, points representing different months largely overlapped, especially in the case of samples collected from vegetated sediments. Points representing samples collected from bare sand were much more dispersed, and the different months were better separated.

**Figure 7:**
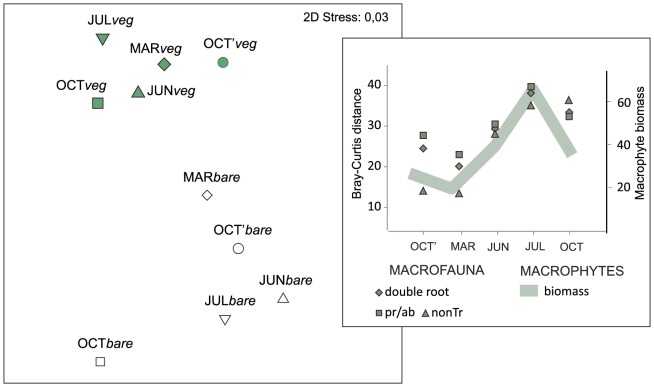
Magnitude of difference in macrofauna community composition between two bottom types in different seasons. nMDS of Bray-Curtis similarities among centroids for groups of samples collected in different months/bottom types (*veg* - vegetated bottom, *bare* - bare sand). Right plot presents Bray-Curtis distances between centroids representing the fauna collected from two bottom types plotted against total macrophyte biomass [g dw m^−2^] in 5 months. Macrobenthic dissimilarity calculated based on: presence/absence data (pr/ab), double root transformed, and untransformed data (nonTr).

The significant interaction of BtxMt (Table 2) suggested that the effects of vegetation on macrobenthic composition might vary across the five months studied. Pairwise comparisons of samples collected from two bottom types performed separately for each month documented significant effects in all months for double root and nonTR data, while for pr/ab data, significant effects were observed for all months except March. In all cases (pr/ab, double root transformed, nonTR data), the distance between the samples collected from the two bottom types changed seasonally, and this temporal pattern was similar to that of macrophyte vegetation characteristics in that the minimum was in March and the maximum was in July (Figure 7).

DISTLM analyses confirmed significant effects of all seven environmental variables on macrobenthic community structure (Table 3). DISTLM results indicated that bottom type explained almost 40% of the variation observed in macrofauna communities. Seven variables (Bt, Mt, St, POC, mean grain size, fine sand, chla) were included in the best fitting model, and together explained 61% of total variation. However, only four variables were statistically significant in the model, namely the three categorical variables and POC.

**Table 3:** Results of DISTLM procedure for fitting environmental variables to macrobenthic composition.

Marginal tests			Sequential tests			
variable	psF	R^2^	variable	psF	R^2^	cumR^2^
month	4.6**	0.16	bottom type	38.0**	0.28	0.28
site	15.5**	0.14	month	7.0**	0.16	0.44
bottom type	38.0**	0.28	site	30.3**	0.14	0.58
POC/N	2.3	0.02	POC	2.4*	0.01	0.59
POC	8.1**	0.08	mean grain size	1.9	0.01	0.60
Chla	9.8**	0.09	fine sand	1.7	0.01	0.61
mean grain size	4.8**	0.05	Chla	1.0	0.00	0.61
fine sand	5.2**	0.05				
δ13C	5.2**	0.05				

Analyses based on double root transformed macrofaunal data of species abundances in samples. Significant effects: * P<0.05; ** P<0.001.

In the vegetated bottom, macrophyte biometrics explained from 10 (density) to 27% (species richness) of variation in macrozoobenthic community characteristics (Table 4). None of the macrophyte biometrics was identified as significant in the model for macrofauna density. Two variables were included in the best fitting model for macrofauna biomass (explaining together 18%) with algal biomass as the only significant variable. Seagrass above ground biomass was the only significant variable among the four variables identified by the model as best fitting to macrofauna species richness variability. Four macrophyte vegetation biometrics were included in the model developed for macrofauna composition with two variables (seagrass above ground biomass and algal biomass) significant at P<0.001.

**Table 4:** Results of DISTLM procedure for fitting macrophyte cover biometrics to macrobenthic community characteristics for samples collected in vegetated sediments.

Marginal tests			Sequential tests			
variable	psF	R^2^	variable	psF	R^2^	cumR^2^
MACROFAUNA DENSITY						
algal biomass	1.6	0.03	algal biomass	1.6	0.03	0.03
seagr. shoot density	0.1	0.00	seagr. shoot density	3.5	0.07	0.10
seagr. ab. ground biomass	0.0	0.02				
total macrophyte biomass	0.1	0.00				
algal species richness	1.0	0.02				
MACROFAUNA BIOMASS						
algal biomass	8.8*	0.16	algal biomass	8.8*	0.16	0.16
seagr. shoot density	3.5	0.07	seagr. shoot density	1.4	0.02	0.18
seagr. ab. ground biomass	5.5*	0.10				
total macrophyte biomass	5.5*	0.10				
algal species richness	6.7*	0.12				
MACROFAUNA SPECIES RICHNESS						
algal biomass	3.4	0.07	seagr. ab. ground biomass	10.5*	0.18	0.18
seagr. shoot density	3.8	0.07	algal species richness	2.9	0.05	0.23
seagr. ab. ground biomass	10.5*	0.18	total macrophyte biomass	1.3	0.02	0.25
total macrophyte biomass	5.9*	0.11	algal biomass	1.5	0.02	0.27
algal species richness	1.2	0.02				
MACROFAUNA COMPOSITION						
algal biomass	5.0**	0.09	seagr. ab. ground biomass	5.1**	0.10	0.10
seagr. shoot density	4.5**	0.08	algal biomass	4.3**	0.07	0.17
seagr. ab. ground biomass	5.1**	0.10	algal species richness	1.3	0.02	0.19
total macrophyte biomass	4.5**	0.09	total macrophyte biomass	1.2	0.03	0.22
algal species richness	4.6**	0.09				

Macrofauna analysed in terms of density, biomass, species richness and species composition (based on double root transformed species abundances in samples data). Macrophyte cover biometrics include total macrophyte biomass, algal biomass, algal species richness, seagrass shoot density (seagr. shoot density) and seagrass above ground biomass (seagr. ab. ground biomass). Significant effects: * P<0.05, **P<0.001.

SIMPER analyses of double root transformed data identified nine species of cont% equal to or higher than 4%. These species altogether contributed to about 45% of the total dissimilarity between vegetated and bare sediments (Table 5). Ten species contributed at least 4% to total dissimilarity between the two bottom types (53% in total) when pr/ab data were analyzed (Table 6). *Theodoxus fluviatilis*, *Idotea balthica*, *Idotea chelipes*, *Mytilus edulis*, and Chironomidae larvae were identified by SIMPER as discriminating the two bottom types in both analyses; they occurred both with higher frequencies and higher densities on vegetated bottoms. None of the discriminating species was found only in samples collected from one bottom type. For most species, higher average densities were recorded on vegetated bottoms, with only *Macoma balthica* occurring with higher numbers on bare sand.

**Table 5:** Species responsible for discrimination of macrofauna between two bottom types, as indicated by the SIMPER procedure based on double-root transformed data.

	SIMPER		JAS		RM	
	Diss/SD	Cont%	bare	veg	bare	veg
*Theodoxus fluviatillis*	1.8	6.2	2.4	17.5	0.8	12.7
*Idotea chelipes*	2.2	6.0	0.1	11.3		3.6
*Mytilus edulis*	2.0	5.0	0.6	5.6	0.2	3.4
*Hydrobia *sp.	1.2	5.0	384.4	410.2	609.6	807.5
*Idotea balthica*	1.8	5.0	0.1	6.3		3.4
Chironomidae larvae	1.5	4.8	3.8	10.5	0.6	10.6
*Cyathura carinata*	1.3	4.5	18.8	13.4	0.5	1.8
*Gammarus *sp. juv	1.4	4.0	4.9	16.2	6.2	10.8
*Pygospio elegans*	1.2	4.0	56.3	27.7	8.4	15.1

Diss/SD – average contribution to overall dissimilarity divided by standard deviation, Cont% - percentage contribution to total dissimilarity. Mean densities [ind. 0.1 m^−2^] in groups of samples defined by bottom type (bare, veg) and station (RM, JAS) are presented. Only species of cont% equal or higher than 4 are listed.

**Table 6:** Species responsible for discrimination of macrofauna between two bottom types, as indicated by the SIMPER procedure, based on presence/absence data.

	SIMPER		JAS		RM	
	Diss/SD	Cont%	bare	veg	bare	veg
*Idotea chelipes*	2.25	6.98	8	100	0	76
*Idotea balthica*	2.00	6.90	12	76	0	96
*Mytilus edulis*	1.73	6.49	24	96	16	92
Bryozoa nd	1.42	5.79	4	60	4	80
*Theodoxus fluviatillis*	1.23	5.19	40	100	32	80
Chironomidae larvae	1.11	4.78	44	88	40	92
*Gammarus oceanicus*	1.10	4.45	8	68	0	44
*Corophium* sp. juv.	0.98	4.18	72	72	36	28
*Marenzelleria neglecta*	0.98	4.15	84	64	16	28
*Lymnaea peregra*	0.96	4.00	32	76	16	20

Diss/SD - average contribution to overall dissimilarity divided by standard deviation, Cont% - percentage contribution to total dissimilarity. Mean frequencies of occurrence [%] in groups of samples defined by bottom type (bare, veg) and station (JAS, RM) are presented. Only species of cont% equal or higher than 4 are listed.

## Discussion

This is the first study of natural seagrass and bare sediment communities that provides evidence that the persistence and magnitude of the impact of macrophyte cover on zoobenthos can be seasonally dependent. The significant positive effects of macrophyte vegetation on invertebrate density and biomass were recorded only in June, July, and October, which are the months when the seagrass canopy is relatively well developed. The strongest contrasts in macrofaunal characteristics between vegetated and unvegetated sediments were observed in July and coincided with the maximum peak in all quantitative characteristics of macrophyte vegetation – seagrass biomass, shoot density, and the biomass and diversity of other associated macrophytes. The vegetation effects on faunal species richness, diversity, and composition persisted throughout the year, but the magnitude of these effects varied seasonally in parallel with the pattern of macrophyte cover seasonal development. Again, the strongest effects (i.e. largest contrasts between the two habitats) were documented in July.

Season-related differences in the effects of seagrass cover on fauna are suggested by Pranovi et al. [30], who compared faunal density, biomass, and diversity between bare sand and a plot with experimental transplants of *Cymodocea nodosa* in the Lagoon of Venice. They found no significant difference between these two treatments in March, while significantly higher standing stocks and diversity of the fauna in the vegetated plot were noted in September, when seagrass shoot density was two times and leaf biomass was seven times higher than in early spring. A few studies conducted in natural seagrass beds also showed that seasonal changes in the quantitative characteristics of faunal assemblages mirrored seasonal variability in seagrass vegetation. For example, maximum values of mollusc species richness and abundance in Mediterranean *Posidonia oceanica* meadows occurred in summer months and coincided with the peaks in seagrass shoot density and leaf length [31], [32], [33], similar observations were reported for mollusc communities inhabiting the green algae *Caulerpa prolifera* meadow [34]. On the other hand, Vonk et al [35] shows that in tropical subtidal seagrass meadows, where the plant biomass does not vary seasonally, the seasonal variation of the fauna is relatively small, much less pronounced that the spatial variability related to the differences in the vegetation biometrics within the meadow. Ecosystem engineering impact depends on the density of the engineering organism, the relationship of which can be non-linear. Moreover, engineering effects can be only detectable above a given threshold level of the engineer's density [12]. In the present study no effect of vegetation on zoobenthic density or biomass could actually be detected in months when macrophyte biomass was equal to or lower than 20 g dw m^−2^. Several studies report that the engineering effects of seagrass are density dependent. Gacia et al. [36] show that sea grass canopies slowed current velocities with intensities proportional to the canopy height of the plants. Shoot density influenced the amount of silt and organic matter in sediments in *Z. marina* beds off the Isles of Scilly [37]. The dependence of invertebrate density and diversity on seagrass shoot density and/or biomass is documented, for example, in *Z. marina* meadows off southwest England for both infaunal [38] and epifaunal communities [5]. In the present study, when only vegetated bottom was considered, faunal community characteristics were also significantly related to different biometrics of macrophyte vegetation (describing both plant and algal components of the meadow).

Bostrom & Bonsdorff [6] list a number of factors that can differentiate the faunal communities associated with seagrass beds and bare sand. They stated that *Zostera* beds had higher habitat complexity, higher food availability, higher organic carbon content, lower flow velocity, finer sediments, higher shelter, lower predation, lower competition, enhanced deposition, and higher sediment stability. It is usually hard to separate the effects of these possible mechanisms and factors when identifying differences between vegetated and bare sands [39]. Seasonal change in the magnitude of differences in faunal communities between sea grass beds and bare sediments in the Puck Bay cannot be explained by the modification of sediment or organic content by plant vegetation. The mean grain size, POC concentration, and δ^13^C differed significantly between the bare sand and vegetated bottom at both stations, but the magnitude of these differences remained stable throughout the year and did not follow seasonal variability in seagrass biomass [21]. The DISTLM procedure identified a number of sediment characteristics (e.g., POC content, fine sediments) as significant drivers of zoobenthic community variability, but R^2^ values indicated their contributions were rather small. Herkul & Kotta [22] studied the effects of the experimental removal of *Z. marina* on sediment characteristics and faunal communities in the northern Baltic Sea. They found sea grass removal had a moderate effect on sediment granulometry (decreased fine grain size fraction), organic matter content, and macrofaunal communities (decreased density and diversity). However, their experiments also included the artificial addition of sand to the seagrass meadow sediments, and this manipulation had no significant effect on the associated macrofauna. These results corroborate our notion that the modification of sediment characteristics is of lesser importance for seagrass meadow macrofauna than other factors, most probably the habitat complexity provided by the vegetation.

Seasonal changes in *Zostera marina* were the most pronounced, but seagrass is not the only phytal element that changed seasonally in the studied meadows. Both the diversity and biomass of other macrophytes changed alongside eelgrass cover seasonal development. In winter, *Z. marina* was the only component of the vegetation, while in summer it was accompanied by six other macrophyte species representing a variety of morphological forms, which together comprised a considerable percentage of the total macrophyte biomass. Habitat structural complexity, which is a key characteristic of seagrass meadows, is produced by a three-dimensional net of interlacing stems, leaves, roots, and rhizomes with a number of gaps and crevices providing a variety of microhabitats to marine infaunal and epifaunal invertebrates [3]. In the Baltic Sea in summer, habitat structural complexity increases largely from increased seagrass biomass (quantitative change), but it also increases from the presence of additional structural components provided by other macrophytes (qualitative change). Increased complexity in seagrass meadow vegetation has been shown to positively influence faunal density and diversity in a number of *in situ* observations and experimental surveys [3], [40], [41]. The seasonal variability in epifaunal density and diversity in seagrass meadows off Florida was related to both increased seagrass biomass and to the occurrence of drift algae, an additional component of habitat complexity, in the summer season [42]. The high total architectural complexity of seagrass habitats, which are produced by the mosaic formed of all structural components, is beneficial to macrofauna as it provides both additional microhabitat niches and protection from predation. Based on their research in Swedish and Finish seagrass beds, Baden et al. [43] report that predation pressure in the Baltic Sea was very low and had no effects on the biomass of mesograzers associated with seagrass. However, the biomass and trophic impact of small benthivorous fish (*Platichthys flesus, Anguilla anguilla, Pomatoschistus minutus, P. microps, Neogobius melanostomus, Zoarces viviparous, Rutilus rutilus*) in the Puck Bay is much higher than in the northern Baltic Sea coastal areas [44] and the protective effects of macrophyte cover could also be more important in this area.

*Zostera* is not an important direct provider of organic carbon to macrobenthic invertebrates in the Baltic Sea benthic system. In Swedish Baltic Sea coastal waters, Jephson et al. [4] identified only two species that consume seagrass tissues – *Rissoa* sp. and *Theodoxus fluviatilis*, and only the former occurs in the Puck Bay. On the other hand, they did identify a number of grazers that depend on the epiphyte algae that accompany *Z. marina* in seagrass meadows. Seagrasses are rarely treated as preferred food, mostly because of their low nitrogen and high cellulose content as well as the presence of condensed tannins and phenolic acids that can be toxic to herbivores [45]. Epiphytes are identified as the most important food source for fauna associated with seagrass in a number of studies [45], [46], [47]. Summer increases in seagrass leaf length and shoot density, which increases the overall surface area of above-ground seagrass organs in the Puck Bay, increases the substrate available for epiphyte colonization. We can expect that epiphyte biomass follows this trend, and that the increased amounts of organic carbon stored in the epiphytes support higher densities of invertebrate grazers in the summer season.

The differences in macrobenthic community composition between vegetated and unvegetated sediments in the Puck Bay were produced by shifts in the dominance of co-occurring species rather than a complete change in species composition. Bivalve molluscs that are important components of the Puck Bay macrozoobenthos in terms of both abundance and biomass occurred at different abundances in seagrass meadows and on bare sand. *Macoma balthica* was the only dominant species that occurred with higher densities on bare sand. In a settlement experiment in the Archipelago Sea, Bostrom et al. [48] show that *Z. marina* vegetation can actually inhibit the settlement of the larvae of the bivalves *M. balthica* and *Cerastoderma glaucum*. They also reported that post-settlement transportation of bivalves by resuspension events and secondary settlements of larger individuals were much more common on bare sands and seagrass patches than in continuous *Z. marina* vegetation. These effects were particularly strong for *M. balthica* [48]. In contrast to *Macoma*, the mussel *Mytilus edulis* occurs almost exclusively in seagrass beds, and seagrass vegetation has been shown to collect drifting clumps of mussels, and, thus, facilitate the post-recruitment settlement of *Mytilus* populations [49]. On the other hand, mussels can fertilize seagrass shoot growth through the biodeposition of organic material via feces and pseudofeces [50]. The gastropod *Theodoxus fluviatilis* was among the species that most strongly discriminated the two communities, both with regard to abundances (analyses of double root transformed data) and frequencies of occurrence (presence/absence data). The strong preference of *Th. fluviatilis* for seagrass beds is not surprising, as this species can use seagrass tissue as a direct food source [4]. A number of isopod (*Idotea chelipes, Idotea balthica*) and amphipod (*Gammarus oceanicus, Gammarus salinus, Gammarus zaddachi*) species were important components of the seagrass communities in the Puck Bay. These are small mesograzers that feed on seagrass epiphytes [4], [51] and good swimmers that can actively disperse in search of vegetated substrates [52].

The temporal and spatial variability of macrozoobenthos was higher on bare sand than on vegetated bottoms. This was indicated by the larger dispersion of bare sand samples both on PCO ordination, and on CAP ordination plotted along the strongest gradients produced by months and stations (Figure 6). This pattern could have been produced by the protective and stabilizing effects of macrophyte cover. Seagrass canopies slow down near-bottom water velocities with intensities proportional to the canopy height of the plants [33]. Thus, seagrass vegetation can mitigate the effects of extreme events such as storms and strong winds that are very common in this part of the Baltic Sea. Bostrom & Bonsdorf [53], in an experimental study of a succession in Baltic Sea sediments, observe that a single event of strong wind could destroy communities on bare sand, while it had little impact on a parallel community developing on a vegetated bottom. Reusch & Chapman [49] also reported that the storm-driven dislodgement of mussels was much more severe on bare sands than in seagrass beds; this is further support for the notion that macrophyte cover provides natural protection against environmental disturbance.

## Conclusions

In this study we have demonstrated that the effects of macrophytes on associated benthic communities in the southern Baltic Sea are seasonally depended. The positive effects of macrophyte cover on the univariate and multivariate characteristics of macrobenthic fauna were either weaker or could not be detected in late fall and early spring when macrophyte vegetation was least developed in terms of both biomass and taxonomic diversity. These findings indicate that in temperate, clearly seasonal systems the assessment of the ecosystem engineer impact cannot be based solely on observations performed in one season only. The common notion that macrophyte cover has a strong influence on benthic fauna in marine coastal habitats is actually mostly based on surveys performed in summer when macrophyte cover is best developed. Thus, the effects of macrophytes can be overestimated to some extent. More studies of seagrass systems performed in other seasons, especially in winter, are strongly recommended if we are to better understand the actual role of macrophyte vegetation in structuring macrozoobenthic communities.

## Supporting Information

Table S1 **Taxonomic composition and densities of taxa in samples collected at two stations, on two bottom types and in five months.** Average values for replicate samples collected at the same stations, bottom types (veg - vegetated bottom, unveg - bare sands) and months are presented.
(XLSX)

## References

[1] Jones CG, Lawton JH, Shachak M (1994) Organisms as ecosystem engineers. Oikos 69: 373–386.

[2] Hemminga MA, Duarte CM (2000) Seagrass Ecology. Cambridge: Cambridge University Press. 289 p.

[3] Gartner A, Tuya F, Lavery PS, McMahon K (2013) Habitat preferences of macroinvertebrate fauna among seagrasses with varying structural forms. J Exp Mar Biol Ecol 439: 143–151.

[4] Jephson T, Nystrom P, Moksnes P-O, Baden SP (2008) Trophic interactions in *Zostera marina* beds along the Swedish coast. Mar Ecol Prog Ser 369: 63–76.

[5] Attrill MJ, Strong JA, Rowden AA (2000) Are macroinvertebrate communities influenced by seagrass structural complexity? Ecography 23: 144–121.

[6] Bostrom C, Bonsdorff E (1997) Community structure and spatial variation of benthic invertebrates associated with *Zostera marina* (L.) beds in the northern Baltic Sea. J Sea Res 37: 153–166.

[7] Bearley A, Wells FE (2000) Invertebrate fauna in seagrasses on Success Bank, Western Australia. Biol Mar Medit 7: 199–202.

[8] Fredriksen S, Backer A, Bostrom C, Christie H (2009) Infauna from *Zostera marina* L. meadows in Norway. Differences in vegetated and unvegetated areas. Mar Biol Res 6: 189–200.

[9] Gambi MC, Conti G, Bremec CS (1998) Polychaete distribution, diversity and seasonality related to seagrass cover in shallow soft bottoms of the Tyrrhenian Sea (Italy). Sci Mar 62: 1–17.

[10] Berkenbusch K, Rowden AA, Probert PK (2000) Temporal and spatial variation in macrofauna community composition imposed by ghost shrimp *Callianassa filholi* bioturbation. Mar Ecol Prog Ser 192: 249–257.

[11] Jones CG, Lawton JH, Shachak M (1997) Positive and negative effects of organisms as physical ecosystem engineers. Ecology 78: 1946–1957.

[12] Harley CDG, O'Riley JL (2011) Non-linear density-dependent effects of an intertidal ecosystem engineer. Oecologia 166: 531–541. 21170751 10.1007/s00442-010-1864-1

[13] Fortino K (2006) Effect of season on the impact of ecosystem engineers in the New River, NC. Hydrobiologia 559: 463–466.

[14] Hasegawa N, Hori M, Mukai H (2008) Seasonal changes in eelgrass functions: current velocity reduction, prevention of sediment resuspension, and control of sediment-water column nutrient flux in relation to eelgrass dynamics. Hydrobiologia 596: 387–399.

[15] Duarte CM, Fourqurean JW, Krause-Jensen D, Olesen B (2006) Dynamics of seagrasses stability and change. In: Larkum AWD, editor. Seagrasses: Biology, Ecology and Conservation. Dordercht: Springer. pp. 271–294.

[16] Duarte C (1989) Temporal biomass variability and production/biomass relationships of seagrass communities. Mar Ecol Prog Ser 51: 269–276.

[17] Fourqurean JW, Willsie A, Rose CD, Rutten LM (2001) Spatial and temporal pattern in seagrass community composition and productivity in South Florida. Mar Biol 138: 341–354.

[18] Guidetti P, Lorenti M, Buia MC, Mazzella L (2002) Temporal dynamics and biomass partitioning in three Adratic seagrass species: *Posidonia oceanica, Cymodocea nodosa, Zostera marina* . Mar Ecol 23: 51–67.

[19] Van Katwijk MM, Bos AR, Kennis P, de Vries R (2010) Vulnerability to eutrophication of a semi-annual life history: a lesson learnt from an extinct eelgrass (*Zostera marina*) population. Biol Conserv 143: 248–254.

[20] Van Lent F, Vershuure JM (1994) Intraspecific variability of Zostera marina L., (eelgrass) in the estuaries and lagoons of the southwestern Netherlands, I. Population dynamics. Aquat Bot 48: 31–58.

[21] Jankowska E, Włodarska-Kowalczuk M, Kotwicki L, Balazy P, Kuliński K (2014) Seasonality in vegetation biometrics and its effects on sediment characteristics and meiofauna in Baltic seagrass meadows, Estuar Coast Shelf S. 139: 159–170.

[22] Herkul K, Kotta J (2009) Effects of eelgrass (*Zostera marina*) canopy removal and sediment addition on sediment characteristics and benthic communities in the Northern Baltic Sea. Mar Ecol 30: 74–82.

[23] Nowacki J (1993) The morphometry of the Gulf. In: Korzeniewski K, editor. Puck Bay. Gdańsk: Fundacja Rozwoju Uniwersytetu Gdańskiego. pp. 71–79.

[24] Kruk-Dowgiałło L (1998) Phytobenthos as an indicator of the state of environment of the Gulf of Gdansk. Oceanological Studies 4: 105–121.

[25] Gic-Grusza G, Kryla-Staszewska L, Urbanski J, Warzocha J, Weslawski JM (2009) Atlas of Polish marine area bottom habitats: environmental valorization of marine habitats. Gdynia: Broker-Inowacji. 179 p.

[26] Dalsgaard T, Nielsen LP, Brotas V, Viaroli P, Underwood G et al. (2000) Protocol handbook for NICE - Nitrogen Cycling in Estuaries: a project under the EU research programme: MAST III. Silkeborg: National Environmental Research Institute. 62 p.

[27] Folk RL, Ward WC (1957) Brazos River bar: a study in the significance of grain size parameters. J Sediment Petrol 27: 3–26.

[28] Anderson MJ, Gorley RN, Clarke KR (2008) PERMANOVA for PRIMER: guide to software and statistical methods. Plymouth: PRIMER–E Ltd. 214 p.

[29] Clarke KR (1993) Non-parametric multivariate analyses of changes in community structure. Aust J Ecol 18: 117–143.

[30] Pranovi F, Curiel D, Rismondo A, Marzocchi M, Scattolin M (2000) Variations of the macrobenthic community in a seagrass transplanted are of the Lagoon of Venice. Sci Mar 64: 303–310.

[31] Carmen Arroyo MdSC, Rueda JL, Gofas S (2006) Temporal changes of mollusc populations from a *Zostera marina* bed in southern Spain (Alboran Sea) with biogeographic considerations. Mar Ecol 27: 417–430.

[32] Urra J, Ramirez AM, Marina P, Salas C, Gofas S, et al (2013) Highly diverse molluscan assemblages of *Posidonia oceanica* meadows in northwestern Alboran Sea (W Mediterranean): seasonal dynamics and environmental drivers. Estuar Coast Shelf S 117: 136–147.

[33] Rueda JL, Salas C (2008) Molluscs associated with a subtidal *Zostera marina* L. bed in southern Spain: linking seasonal changes of fauna and environmental variables. Estuar Coast Shelf S 79: 157–167.

[34] Rueda JL, Salas C (2003) Seasonal variation of a molluscan assemblage living in a *Caulerpa prolifera* meadow within the inner Bay of Cadiz (SW Spain). Estuar Coast Shelf S 57: 909–918.

[35] Vonk JA, Christianen MJA, Stapel J (2010) Abundance, edge effect, and seasonality of fauna in mixed-species seagrass meadows in southwest Sulawesi, Indonesia. Mar Biol Res 6: 282–291.

[36] Gacia E, Granata TC, Duarte CM (1999) An approach to measurement of particle flux and sediment retention within seagrass (*Posidonia oceanica*) meadows. Aquat Bot 65: 255–268.

[37] Bowden DA, Rowden AA, Attrill MJ (2001) Effect of patch size and in-patch location on the infaunal macroinvertebrate assemblages of *Zostera marina* seagrass beds. J Exp Mar Biol Ecol 259: 133–154. 11343709 10.1016/s0022-0981(01)00236-2

[38] Webster PJ, Rowden AA, Attrill MJ (1998) Effect of shoot density on the infaunal macro-invertebrate community within a *Zostera marina* seagrass bed. Estuar Coast Shelf S 47: 351–357.

[39] Bouma TJ, Ortells V, Ysebaert T (2009) Comparing biodiversity effects among ecosystem engineers of contrasting strength: macrofauna diversity in *Zostera noltii* and *Spartina anglica* vegetations, Helgol Mar Res. 63: 3–18.

[40] Stoner AW, Lewis FG (1985) The influence of quantitative and qualitative aspects of habitat complexity in tropical seagrass meadows. J Exp Mar Biol Ecol 94: 19–40.

[41] Sirota L, Hovel KA (2006) Simulated eelgrass *Zostera marina* structural complexity: effects of shoot length, shoot density, and surface area on the epifaunal community of San Diego Bay, California, USA. Mar Ecol Prog Ser 326: 115–131.

[42] Gore RH, Gallaher EE, Scotto LE, Wilson KA (1981) Studies on decapod Crustacea from the Indian River region of Florida. Estuar Coast Shelf S 12: 485–508.

[43] Baden S, Bostrom C, Tobiasson S, Arponen H, Moksnes P-O (2010) Relative importance of trophic interactions and nutrient enrichment in seagrass ecosystems: a broad-scale field experiment in the Baltic-Skagerrak area. Limnol Oceanogr 55: 1435–1448.

[44] Tomczak MT, Muller-Karulis B, Jarv L, Kotta J, Martin G, et al (2009) Analyses of trophic networks and carbon flows in south-eastern Baltic Coastal ecosystems. Prog Oceanogr 81: 111–131.

[45] Bologna PAX, Heck KL (1999) Macrofaunal associations with seagrass epiphytes. Relative importance of trophic and structural aspects. J Exp Mar Biol Ecol 242: 21–39.

[46] Kitting CL, Fry B, Morgan MD (1984) Detection of inconspicuous epiphytic algae supporting food webs in seagrass meadows. Oecologia 62: 145–149. 28310706 10.1007/BF00379006

[47] Heck KL, Valentine JF (2006) Pant-herbivore interactions in seagrass meadows. J Exp Mar Biol Ecol 330: 420–436.

[48] Bostrom C, Tornroos A, Bondsdorff E (2010) Invertebrate dispersal and habitat heterogeneity: expression of biological traits in a seagrass landscape. J Exp Mar Biol Ecol 390: 106–117.

[49] Reusch TBH, Chapman ARO (1995) Storm effects on eelgrass (*Zostera marina* L.) and blue mussel (*Mytilus edulis* L.) beds. J Exp Mar Biol Ecol 192: 257–271.

[50] Reusch TBH, Chapman ARO, Groger JP (1994) Blue mussel *Mytilus edulis* do not interfere with eelgrass *Zostera marina* but fertilize shoot growth through biodeposition. Mar Ecol Prog Ser 108: 265–282.

[51] Leidenberger S, Harding K, Jonsson PR (2012) Ecology and distribution of the isopod genus *Idothea* in the Baltic Sea: key species in a changing environment. J Crustacean Biol 32: 359–381.

[52] Bostrom C, Mattila J (1999) The relative importance of food and shelter for seagrass associated invertebrates- a latitudinal comparison of habitat choice by isopod grazers. Oecologia 120: 162–170. 28308048 10.1007/s004420050845

[53] Bostrom C, Bonsdorf E (2000) Zoobenthic community establishment and habitat complexity - the importance of seagrass shoot-density, morphology and physical disturbance for faunal recruitment. Mar Ecol Prog Ser 205: 123–138.

